# Small Particle DEBIRI TACE as Salvage Therapy in Patients with Liver Dominant Colorectal Cancer Metastasis: Retrospective Analysis of Safety and Outcomes

**DOI:** 10.3390/curroncol29010020

**Published:** 2022-01-06

**Authors:** Nicolas Voizard, Tiffany Ni, Alex Kiss, Robyn Pugash, Michael Jonathon Raphael, Natalie Coburn, Elizabeth David

**Affiliations:** 1 Odette Cancer Centre, Sunnybrook Health Sciences Centre, Toronto, ON M4N 3M5, Canada; nicolas.voizard@gmail.com (N.V.); tiffany.ni@mail.utoronto.ca (T.N.); alex.kiss@ices.on.ca (A.K.); robyn.pugash@sunnybrook.ca (R.P.); michaelj.raphael@sunnybrook.ca (M.J.R.); natalie.coburn@sunnybrook.ca (N.C.); 2 Temerty Faculty of Medicine, University of Toronto, Toronto, ON M5S 1A8, Canada

**Keywords:** transarterial chemoembolization, colorectal cancer, drug eluting beads with irinotecan

## Abstract

The aim of this study was to examine the safety and efficacy of 40 µm and 75 µm calibrated irinotecan-eluting beads (DEBIRI-TACE) for the treatment of colorectal cancer metastases. We conducted a retrospective review of 36 patients with unresectable liver metastases from colorectal cancer who were treated with DEBIRI-TACE between 2017 to 2020. Patients who received at least one session of DEBIRI were included in our analysis. A total of 105 DEBIRI sessions were completed. 86% of patients (*n* = 31) underwent one round of treatment, 14% of patients (*n* = 5) underwent two distinct rounds of treatment. The majority of patients were discharged the next day (92%, *n* = 33 patients) with no 30-day post-DEBIRI mortality. Five high-grade adverse events occurred, including longer stay for pain management (*n* = 2), postembolization syndrome requiring readmission (*n* = 2), and liver abscess (*n* = 1). The average survival from diagnosis of metastatic disease was 33.3 months (range 11–95, median 28). Nine of 36 patients are still alive (December 2020) and have an average follow-up time of 36.8 months from T0 (range 12–63, median 39). Small particle DEBIRI is safe and well-tolerated in the salvage setting, with outcomes comparable to that of larger bead sizes.

## 1. Introduction

Colorectal cancer (CRC) is the third most commonly diagnosed cancer in Canada [1,2]. About one-quarter of CRC are metastatic at the time of diagnosis, and many lower stage cancers will eventually metastasize, most commonly to the liver [3]. Extensive liver metastases resulting in liver failure is a common cause of death in adults with CRC [4,5].

With the use of more aggressive treatment strategies for metastatic CRC, including surgery, neoadjuvant and adjuvant chemotherapy, portal vein embolization, radiation therapy, thermal ablation, transarterial radioembolization (TARE), and chemoembolization (TACE), the mortality rate has decreased significantly in recent years [3]. Twenty to 30% of metastatic patients are candidates for surgical resection, which is the first line of treatment for liver-only metastatic disease. Unfortunately, almost 70% of patients experience disease recurrence at 5-years [6]. The largest single-center investigation on liver transplant (LT) for CRC demonstrated a low 5-year overall survival of 12%, despite restricting LT to node-negative primaries. Retrospectively, they found that micrometastases were present in 15 of 21 patients initially deemed node-negative. This finding suggests that, even with the widest resection margin attainable, the possibility of recurrence from undetected micrometastases is an ever-present threat in CRC [7]. Cure is elusive beyond five years, but control is possible with the myriad of currently available options. If CRC has indeed transformed into a chronic disease, then treatments need to focus on both survival and preserving the functional status of the liver and the patient. Ideally treatments should be easy to repeat and not limit future options.

For non-surgical patients, standard first-line chemotherapy is 5-FU, folinic acid with either irinotecan (FOLFIRI) or oxaliplatin (FOLFOX) [8]. When used in combination with targeted therapy, including epidermal growth factor receptor (EGFR) and vascular endothelial growth factor (VEGF) inhibitors, overall survival improved to 18–29 months [9].

Irinotecan drug-eluting bead (DEBIRI) TACE has been widely used in recent years as salvage therapy and, infrequently, as first or second-line therapy [10]. The lack of consensus on its use is multifactorial. DEBIRI is still relatively novel, with the first human study published in 2012 [11]. Since then, only two randomized controlled trials, consisting of heterogenous patients receiving a wide array of treatment algorithms, have been conducted. These studies compared DEBIRI to conventional chemotherapy and demonstrated a mean survival following DEBIRI of 15.3 and 22 months, respectively [12,13]. 

Smaller drug-eluting beads of 40 µm and 75 µm size became available in Canada in 2017. The use of smaller bead sizes may result in improved tumor penetration resulting in more durable treatment response [14]. Small prospective studies have further shown the use of 40 µm beads to be safe [15], even with concurrent systemic chemotherapy [16].

The purpose of this retrospective study is to analyze the efficacy and safety of 40 µm and 75 µm calibrated beads loaded with irinotecan (DEBIRI) for the treatment of CRC metastases delivered in a single centre since their availability in Canada (2017).

## 2. Materials and Methods

### 2.1. Patient Population

This single-centre retrospective study includes 36 patients (9 women and 27 men) with unresectable liver metastases from CRC treated with DEBIRI between December 2017 and December 2020 at Sunnybrook Health Science Center (SHSC), Toronto, Canada. The study was approved by the local institutional ethics board (Study ID 3437). 

All patients (*n* = 37) with histologically proven metastases from a primary colorectal cancer who had been treated at least once with DEBIRI using 40 µm or 70 µm calibrated beads were deemed eligible. Patients were excluded (*n* = 1) if no histologic proof of primary CRC could be found or if no detail was available in their chart regarding the prior history of chemotherapy, surgery, radiation therapy, ablation, or embolization.

Patients were selected for DEBIRI treatment after tumor board review. According to indications from the European Society for Medical Oncology and National Comprehensive Cancer network, patients treated with DEBIRI were selected based on the presence of non-resectable disease, disease refractory to conventional lines of chemotherapy, contraindication to surgery or systemic chemotherapy or non-tolerance of systemic chemotherapy with Eastern Cooperative Oncology Group (ECOG) performance status of two or lower, normal hematological values, ALT and GGT < 3 times, and total bilirubin < 2 times the upper limit of normal [10,17]. Details are outlined in Table 1. For procedure details and data collection and definitions, see Appendix A.

Adverse events (AE) were collected based on chart review and not on patient questioning. Postembolization syndrome (PES) was not considered an AE as it was commonly encountered. AE terminology was set forth by the Cancer Therapy Evaluation Program Common Terminology Criteria for Adverse Events CTCAE, version 5.0.

### 2.2. Statistical Analysis

Descriptive statistics were calculated for all variables of interest. Bivariate Cox proportional hazards (PH) models were used to assess the impact of variables on overall survival (OS). A multivariable Cox PH model was run on three variables, which was the maximum allowed given the sample size. Prior to analysis, the variables were assessed for multicollinearity (tolerance statistic < 0.4). No evidence of multicollinearity was found. The bivariate and multivariable survival analysis results were reported as hazard ratios and their associated 95% confidence intervals. Statistical significance was set to *p* ≤ 0.05. A Kaplan–Meier curve was created for survival from metastatic disease (T0). All analyses were carried out using SAS Version 9.4 (SAS Institute, Cary, NC, USA).

## 3. Results

The majority of patients in this study had synchronous liver disease at presentation, 75% of patients (*n* = 27) had no neo-adjuvant therapy. Prior to DEBIRI treatments 14% (*n* = 5), 50% (*n* = 18), 31% (*n* = 11), and 6% (*n* = 2) of patients had 0, 1, 2, or >3 systemic lines of treatment, respectively (Table 1.). Targeted therapy (EGFR and/or VEGF inhibitors) was used in 72% of patients (*n* = 26).

Liver resection was performed in 33% (*n* = 12) of patients. Two of these patients underwent two distinct liver resections at 1.4 and 4.7 years apart. Intra-operative ablation concurrent to liver resection was performed in 14% (*n* = 5) patients. 

A total of 105 DEBIRI sessions were completed, 86% of patients (*n* = 31) underwent one round of treatment, and 14% of patients (*n* = 5) underwent two distinct rounds of treatment, each comprising multiple sessions. The average number of sessions was 2.9 (range 1–8, median 3) for all patients, 2.7 (range 1–5, median 3) for patients who underwent only one round, and 4.6 (range 2–8, median 4) for patients who underwent two rounds of DEBIRI treatment.

In addition to surgery, systemic chemotherapy, and DEBIRI, 22% of patients (*n* = 8) underwent one to two sessions of stereotactic body radiation therapy (SBRT), 42% of patients (*n* = 15) underwent at least one ablation session (microwave or radiofrequency) (range 1–7, mean 2.13, median 2). On average, patients underwent a total of 4.6 lines of treatment (range 2–12, median 4.6) after diagnosis of metastatic disease.

The majority of patients were discharged the next day (92%, *n* = 33 patients). Two patients required a 2-day hospitalization after a single session. One patient had a mean length of stay < 1 day, being discharged the same day after several DEBIRI sessions (Table 2).

Fourteen percent of patients (*n* = 5) had Grade 3 AE (Table 2). Two patients had low-grade fever with no source, compatible with PES. One patient was readmitted for drainage of a liver abscess less than two weeks after the first DEBIRI session. 

**Figure 1:**
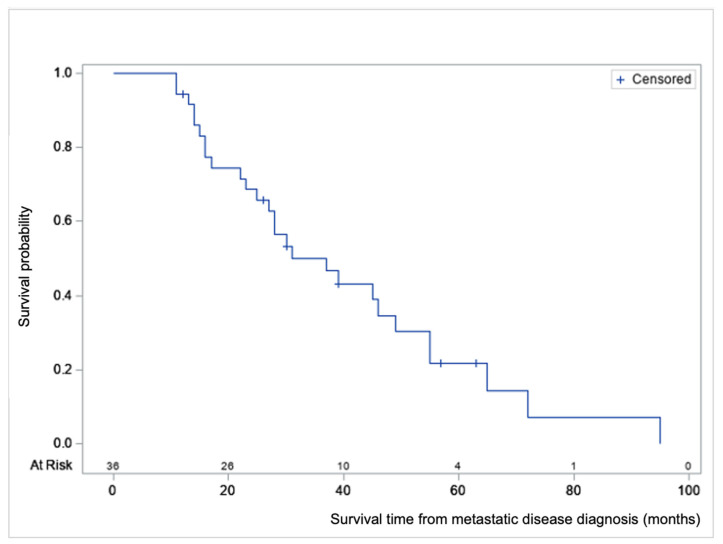
Product-limit survival estimate from metastatic disease diagnosis (T0) with number of subjects at risk, in months.

Survival analyses were performed from T0, diagnosis of metastatic disease (Figure 1, Table 3 and Table 4). The average OS from CRC diagnosis was 39.8 months (range 13–145, median 28). 

The average survival from diagnosis of metastatic disease was 33.3 months (range 11–95, median 28). Nine of the 36 patients are still alive (December 2020) and have an average follow-up time of 36.8 months from T0 (range 12–63, median 39). No 30-day post-DEBIRI mortality was seen. From the first DEBIRI session to death, the average time was 11.6 months (range 2–36, median 11.6).

In univariate analysis, ECOG performance status, total number of lines after T0, time to first DEBIRI, early DEBIRI treatment, and primary resection were found to have a significant effect on OS (Table 4).

Patients with an ECOG of 1 or 2 at first DEBIRI session had a 2.63 higher hazard of death than those with an ECOG of 0 (*p* = 0.03, 95% CI 1.11–6.20). There was a positive correlation between survival and total number lines after metastatic disease (T0) such that each one unit increase in total lines leads to a 0.70 times lower hazard of death (*p* = 0.0007, 95% CI 0.57–0.86).

A negative correlation between survival after T0 and time to first DEBIRI was found such that each additional month before the introduction of DEBIRI leads to a 0.89 times lower hazard of death (*p* = 0.0001, 95% CI, 0.83–0.94). Patients who underwent DEBIRI ≤ 12 months of T0 (*n* = 9) had a statistically significant lower survival with an average of 15.6 ± 4.3 months versus 42.2 ± 20.6 months for those who underwent DEBIRI later (*p* < 0.001). One patient who underwent early DEBIRI is still alive with only 12 months of follow-up.

Resection of primary cancer was shown to provide a 0.32 times lower hazard of death (*p* = 0.02, 95% CI 0.12–0.86). 

Other parameters including KRAS status, carcinoembryonic antigen (CEA) level, site of primary, use of targeted therapy, and prior liver resection did not show any statistically significant role on OS in this study.

In multivariate Cox proportional hazard analysis (Table 4), primary resection and total number of lines after T0 were independent predictors of survival with a lower hazard of death of respectively 0.24 (*p* = 0.03, 95% CI 0.065–0.892) and 0.65 (*p* = 0.0009, 95% CI 0.50–0.84). KRAS mutation status remained a non-significant predictor of survival (*p* = 1.742).

## 4. Discussion

**Figure 2:**
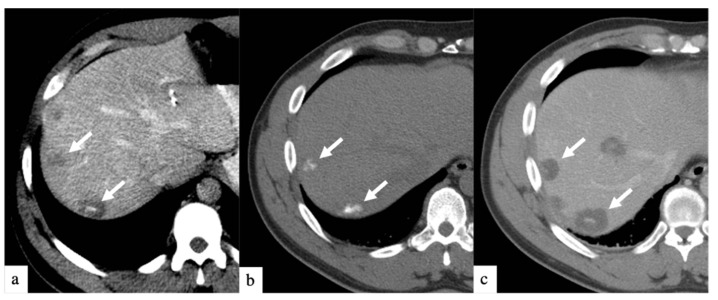
Axial CT images of a patient with history of colorectal cancer who underwent a single small particle DEBIRI TACE session of right lobe. Portal venous phase CT obtained pre-treatment (**a**) shows multifocal liver metastases (arrows); non-enhanced CT obtained 1 h post-treatment (**b**) shows intra-tumoral staining of iodinated contrast trapped by the 40 µm calibrated drug-eluting beads; one-month follow up imaging (**c**) shows increased areas of low attenuation in keeping with devascularization of tumors (arrows). Patient did not undergo a second DEBIRI due to lowering CEA and evidence of response on imaging. One-year follow-up (material not intended for publication) revealed tumoral progression, patient is being considered for second DEBIRI TACE or TARE at time of publication. Note: although not shown, all lesions seen in (**c**) were present on (**a**) and treated.

**Figure 3:**
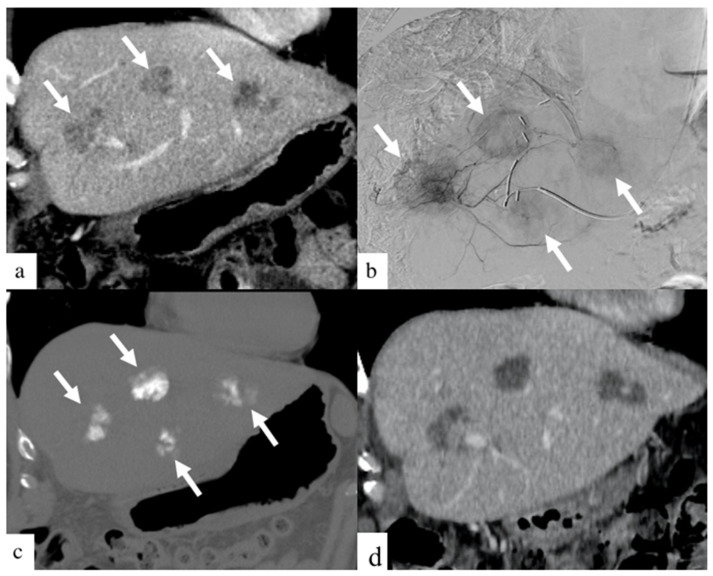
Images of a patient with history of colorectal cancer who underwent a single small particle DEBIRI TACE session of the left lobe. Portal venous phase coronal CT image obtained pre- treatment (**a**) shows three of the four metastases in the left lobe (arrows). Digital subtraction angiographic images obtained through microcatheter (**b**) shows hypervascularity of all four metastases (arrows). Non-enhanced coronal CT image obtained 1 h post-treatment (**c**) shows intra-tumoral staining of iodinated contrast (arrows). Coronal CT image (**d**) of a patient one- month post-DEBIRI treatment.

DEBIRI therapy is known to be safe in patients receiving concurrent systemic chemotherapy [18]. Although prior randomized controlled trials [12,13] and prospective studies [19,20] have shown the safety of treatment with larger beads, few studies have reported the safety profile of smaller beads [14,16]. Smaller bead size has the potential of delivering higher drug concentrations distally into the tumor bed, as depicted in Figure 2 and Figure 3. This retrospective study is, to our knowledge, the largest study evaluating the safety and efficacy of 40 and 75 µm calibrated drug-eluting beads in the treatment of metastatic CRC.

In the present study, the rate of high-grade AE with smaller beads (5/36) was comparable to the 11% described in the literature for larger beads [10] despite its use in a high-risk population with multiple comorbidities and prior treatments. While the high-grade AE required hospitalization or readmission, the majority (4/5) were self-limited, related to pain and fever secondary to postembolization syndrome (PES). One patient developed a hepatic abscess. This patient recovered and was then treated with TARE 12 months post-DEBIRI. Low grade (1 and 2) AE were not collected in this study. There were no postembolization bilomas in this study despite the purported risk of bilomas when using small particles. Small particle embolization has been described in the NET (neuroendocrine population) and is well tolerated. Additionally, based on the hepatic arterial infusion pump (HAIP) program at our hospital, liquid chemotherapy is also relatively well tolerated within a certain range of dose and frequency [21,22]. In addition, TARE with resin spheres has an embolic effect ranging from 20–60 µm [23]. Given these experiences, it is not surprising that embolization with small drug-eluting beads would be well tolerated. 

In this study, patients underwent a median of four total lines of treatment and had a median survival after diagnosis of metastatic disease of 28 months which compares favorably to the median OS of 22 months from CRC diagnosis described by Fiorentini et al. in 2012, who performed DEBIRI using larger 100–300 µm beads [13]. Similarly, a combination of three randomized controlled trials evaluating survival after use of selective internal radiation therapy in combination with chemotherapy has also demonstrated survival of 22.6 months [24]. For comparison, the current median overall survival for patients under conventional systemic therapy is approximately 20 months [25]. 

Systemic chemotherapy, surgery, and ablative therapy impact favorably on survival, but the role of DEBIRI is still debated. Survival analyses demonstrate that patients who were eligible for and who tolerated the most lines of treatment inevitably had the longest survival. These findings reinforce the importance of having many different lines of therapeutic options which can be repeated, are well tolerated, maintain quality of life, and do not limit future treatment options. 

There was a negative correlation between OS and time to DEBIRI and a higher HR of death obtained for patients who were treated earlier in the disease (≤12 months). DEBIRI is presently used as salvage therapy at our institution when all the other lines have failed. An early introduction of DEBIRI implies aggressive disease with unfavorable biology and rapid progression through conventional lines of therapy. Primary cancer was left in situ in 44% of the patients who underwent early DEBIRI versus 11% of late DEBIRI and is a known indicator of poor prognosis [26]. These findings remind us that patients who fail early lines of treatment will likely have a poor response to additional lines of treatment. The test of time is probably the strongest predictor of survival currently available.

Locoregional techniques such as DEBIRI and percutaneous ablation can be repeated as long as there is no significant extrahepatic disease progression, liver function is maintained, and patients have a favorable functional status. Although patients with ECOG 1 and 2 may be candidates for DEBIRI, we found ECOG 0 was a good predictor of longer survival. This is likely multifactorial, supporting that functional status and quality of life are also important factors in overall survival. 

In this small sample, survivals were comparable to trials for large particle DEBIRI and even radioembolization. How DEBIRI should be used, especially as radioembolization becomes more readily available, is even less clear. It may have a role in patients with dose limitations in the liver from prior SBRT treatments. This will require further investigation. In this study, some patients received both chemoembolization and radioembolization and tolerated both well. Again, this underscores the importance of having many lines of treatment available to further lengthen survival. Patients with the longest survivals from multiple treatments often reached a point where disease in the liver stabilized or regressed, but the extrahepatic disease progressed; most commonly, this happened in the lung. The longest survivor in this study eventually succumbed not from liver failure but from respiratory failure due to progressive lung metastases after 12 years of treatment which included every line of therapy available, including radioembolization and DEBIRI.

This study is limited by the small number of patients evaluated, the retrospective design, and the heterogeneity of the patient population. There is also heterogeneity in operator technique resulting in differences in particle dilution, suspension, and rate of delivery. A larger number of patients enrolled within the study and clearly defined delivery parameters for the embolic would enable a more comprehensive detailed analysis. A larger cohort of patients will also allow us to define sub-groups of patients who would most benefit from a particular line of treatment.

## 5. Conclusions

While metastatic CRC is difficult to cure, control is possible, and long-term survival is best achieved when many treatment lines are available for utilization. Even in heavily pretreated patients, DEBIRI was safe and well-tolerated. These results demonstrate that DEBIRI has utility in the salvage setting, although its role in early disease remains unclear. In summary, small particle DEBIRI using 40 and 75 µm drug-eluting beads is safe and provides survival benefits comparable to larger bead sizes.

## Figures and Tables

**Table 1:** Population demographics, primary tumor and metastatic disease characteristics and lines of treatments provided for metastatic colorectal cancer.

Variable	Characteristics	No. Patient (%) or Value
Population		
*n*		36
Age, year		64 ± 13
Gender, *n* (%)	M/F	27 (75%)/9 (25%)
ECOG, *n* (%)	Group 0/group 1/group 2/unknown	18 (50%)/13 (36%)/2 (6%)/3 (8%)
Colorectal Cancer		
Primary Cancer		
Location, *n* (%)	Right-sided/left-sided/multifocal	8 (22%)/27 (75%)/1 (3%)
Primary CRC resection, *n* (%)	Resected/in situ	29 (81%)/7 (19%)
Mutation status, *n* (%)	KRAS mutated/wild-type/unknown	14 (39%)/14 (39%)/8 (22%)
Metastatic Liver Disease		
Presentation, *n* (%)	Synchronous/metachronous	21 (58%)/15 (42%)
Liver dominance, *n* (%)	Liver only/dominant	23 (64%)/13 (36%)
Liver lobe, *n* (%)	Bilobar/unilobar right/unilobar left	34 (94%)/2 (6%)/0 (0%)
Tumoral burden, *n* (%)	<25%/25–50%/50–75%/>75%	10 (28%)/19 (53%)/3 (11%)/3 (8%)
Lines of Treatment		
Conventional systemic therapy		
Neo adjuvant lines, *n* (%)	0/1/2/≥3 lines	27 (75%)/9 (25%)/0 (0%)/0 (0%)
Adjuvant lines before DEBIRI, *n* (%)	0/1/2/≥3 lines	5 (14%)/18 (50%)/11 (31%)/2 (6%)
Adjuvant lines total, *n* (%)	0/1/2/≥3 lines	1 (3%)/14 (39%)/12 (33%)/9 (25%)
Targeted systemic therapy		
EGFR inhibitors, *n*(%)		22 (61%)
VEGF inhibitors, *n*(%)		8 (22%)
EGFR- and VEGF-inhibitors, *n*(%)		4 (11%)
No targeted therapy, *n* (%)		10 (28%)
Surgery		
Liver resection, *n* (%)	1/2 resections	10 (28%)/2 (6%)
Intra-operative ablation, *n* (%)		5 (14%)
Ablation		
Radiation therapy, *n* (%)	1/2 treatments	7 (19%)/1 (3%)
Percutaneous ablation, *n* (%)	1/2/≥3 treatments	6 (17%)/5 (14%)/4 (11%)
DEBIRI		
Rounds of treatments, *n* (%)	1/2 rounds of treatment	31 (86%)/5 (14%)
Total number of sessions, *n* (%)	1/2/3/≥4 sessions	7 (19%)/9 (25%)/7 (19%)/13 (36%)
Early DEBIRI (≤12 months), *n* (%)	Alive/deceased patients	1 (2.8%)/9 (25%)
Mean irinotecan/session, mg		82
Bead size, *n* (%)	variable	17 (47%)/11 (31%)/8 (22%)
Access, *n* (%)	radial/femoral/variable	29 (81%)/5 (14%)/2 (6%)
Total lines after T0 ^1^, *n* (%)	1 line	0 (0%)
	2 lines	8 (22%)
	3 lines	6 (17%)
	4 lines	5 (14%)
	5 lines	3 (8%)
	6 lines	7 (19%)
	7 lines	5 (14%)
	≥8 lines	2 (6%)

^1^ Sum of systemic (convention, targeted), surgical, ablative (radiation, percutaneous) and DEBIRI treatments after metastatic disease (T0).

**Table 2:** Length of stay, readmission and adverse events after DEBIRI.

Variable	No. Patient (%)
Mean Length of Stay	
<1 day, *n* (%)	1 (3%)
=1 day, *n* (%)	33 (92%)
>1 day, *n* (%)	2 (6%)
Readmission <1 month after DEBIRI	
All causes, *n* (%)	5 (14%)
DEBIRI related, *n* (%)	3 (8%)
Adverse events (Grade 3–4)	
Longer length of stay for pain management, *n* (%)	2 (6%)
PES requiring readmission, *n* (%)	2 (6%)
Liver abscess, *n* (%)	1 (3%)

**Table 3:** Time to metastatic disease, survival, and follow-up ^†^.

Variable	Value	No. Patient (%) or Months
Metachronous Presentation		15 (42%)
Time to metastatic disease, months	Median (min, max)	10 (3, 50)
	Mean ± SD	14.1 ± 13.0
Deceased patient, *n* (%)		27 (75%)
OS (from diagnosis), months	Median (min, max)	28 (13, 145)
	Mean ± SD	39.8 ± 29.7
Survival from T0 ^‡^		
All patients, months	Median (min, max)	28 (11, 95)
	Mean ± SD	33.3 ± 21.1
Early DEBIRI ^§^ only, months	Mean ± SD	15.6 ± 4.3
Late DEBIRI only, months	Mean ± SD	42.2 ± 20.6
Time first DEBIRI—death, months	Median (min, max)	10 (2, 36)
	Mean ± SD	11.6 ± 8.8
Alive patient, *n* (%)		9 (25%)
Follow-up time, months	Median (min, max)	39 (12, 63)
	Mean ± SD	36.8 ± 15.8

^†^ Last follow-up 31 December 2020; ^‡^ T0, time of metastatic disease diagnosis; ^§^ Early and late DEBIRI are ≤12 or >12 months.

**Table 4:** Cox regression hazard ratio (HR) in univariate and multivariate analysis for predicting death.

Variable	Univariate Analysis			Multivariate Analysis		
	HR	95% CI	*p*-Value	HR	95% CI	*p*-Value
Age (>60 vs. ≤60)	1.17	0.51 to 2.68	0.7129			
Sex (F vs. M)	0.59	0.22 to 1.60	0.3010			
**ECOG (Group 1 and 2 vs. Group 1)**	**2.63**	**1.11 to 6.20**	**0.0276**			
**Total number of lines after T0**	**0.70**	**0.57 to 0.86**	**0.0007**	**0.65**	**0.504 to 0.837**	**0.0009**
Number chemo lines before DEBIRI	0.77	0.47 to 1.26	0.2996			
**Time to first DEBIRI (months from T0)**	**0.89**	**0.83 to 0.94**	**0.0001**			
**Late (>12 months) vs. early (** **≤** **12 months) DEBIRI**	**31.4**	**6.4 to 154.0**	**<0.0001**			
No use vs. use of VEGF- and/or EGFR-inhibitors	0.63	0.27 to 1.46	0.2793			
Presence KRAS mutation vs. wild-type	1.46	0.57 to 3.72	0.4314	1.742	0.643 to 4.716	0.2747
CEA (>5 ng/mL vs. ≤5 ng/mL)	1.59	0.57 to 4.40	0.3735			
Side of primary CRC (right side or multifocal vs. left)	1.37	0.59 to 3.17	0.4690			
**Primary resection (resected vs. non-resected)**	**0.32**	**0.12 to 0.86**	**0.0235**	**0.241**	**0.065 to 0.892**	**0.0331**
Presentation (metachronous vs. synchronous)	0.62	0.26 to 1.45	0.2674			
Liver resection (resected vs. non-resected)	0.52	0.22 to 1.24	0.1377			
Disease dominance (liver dominant vs. liver only)	1.03	0.44 to 2.41	0.9501			
Number of DEBIRI rounds (2 vs. 1 round)	0.44	0.13 to 1.55	0.2023			

Statistically significant (*p* < 0.05) HR are highlighted in bold.

## Data Availability

The data presented in this study are available on request from the corresponding author. The data are not publicly available due to privacy restrictions.

## Appendix A

**Procedure**

If irinotecan-based chemotherapy was to be given at the time of DEBIRI, it was held prior to treatment. Recent (<1 month) multiphasic CT was required before the first DEBIRI treatment to evaluate tumoral burden, identify feeding vessels, anatomical variants and regions at risk of non-target embolization. All CT examinations conducted were multiphasic CT. A complete round of treatment consists of treating each liver lobe twice, at four weeks intervals, alternating between lobes every two weeks for cases of bilobar disease. The first session generally targeted the most affected lobe; further sessions were performed further spaced apart or in a sublobar fashion depending on tolerance to treatment. CT was not routinely performed between sessions but, if imaging demonstrated disease progression or if the procedure was not tolerated, the remaining sessions were cancelled.

Patients were admitted on the day of the procedure unless prophylactic hydration to protect renal function was needed, in which case they were admitted on the day before. DEBIRI was performed by one of the four experienced interventional radiologists in SHSC, under conscious sedation, using a transradial or distal transradial approach in the vast majority of cases. Femoral access was used if radial access was contraindicated. A 4-5F base catheter was used to obtain non-selective angiograms from the celiac trunk and other visceral branches depending on anatomical variant (e.g., SMA, phrenic). Microcatheter and microwire were used to selectively cannulate segmental branches, perform angiograms and embolize. When oligometastatic disease was present and/or definite tumoral feeders were identified, superselective catheterization was performed to achieve enhanced tumoral coverage.

Technical DEBIRI success was defined as devascularisation on follow-up CT imaging with stability or reduction in the size of marker lesions and no new lesions compared to pre-treatment CT images [27]. Additionally, based on the Choi criteria studies, changes in tumor density was determined by measuring the CT attenuation coefficient, in Hounsfield units [27].

Prior to DEBIRI, irinotecan-HCl (20 mg/mL solution) was loaded on 40 or 75 µm microspheres (Embozene Tandem Microspheres, Boston Scientific, Marlborough, MA, USA) according to the manufacturer’s instructions at 50 mg irinotecan per milliliter (100–150 mg of irinotecan per 2–3 cc syringe depending on tumoral burden). After diluting and homogeneously suspending the loaded microspheres in a mixture of normal saline and contrast mixture (total of 40 to 80 cc depending on vascularity and vessel diameter), the solution was slowly delivered in the targeted territory in small aliquots until near-stagnation in the main trunks. Intra-arterial nitroglycerin and/or calcium channel blockers were occasionally given through the catheter during the procedure depending on angiographic findings. After final angiograms, the catheter and sheath were removed, and patent hemostasis was obtained with a radial band on the wrist or manual compression of the groin.

Patients were observed overnight in the short stay unit and discharged the following day after having been given anti-emetics, analgesics and a course of antibiotics if they were considered at high risk of infection (biliary-enteric anastomosis or sphincterotomy). Patients typically did not have significant post procedural discomfort when using small particles and discharge at 24 h post procedure was common.

**Data Collection and Definitions**

A list of patients with liver metastases from CRC who received DEBIRI embolization with 40 or 75 µm microsphere between 1 December 2017, and 31 December 2020, was generated using picture archiving and communication system (PACS); data were collected by reviewing patient medical records (SunnyCare and Sovera) and imaging.

Data collected from eligible patients included demographic information such as gender, age at DEBIRI, primary cancer site, the reason for referral, pre- and post-procedure imaging, clinical outcomes, and any reported complications. Treatment data pre- and post-embolization for the use of any systemic therapies (including chemotherapy and targeted therapy), surgical resection, radiation therapy, thermal ablation and others were also collected.

Time-0 (T0) was defined as the date of diagnosis of metastatic disease, whether the patient presented first with intra or extrahepatic metastasis.

Survival time from metastatic disease was calculated from T0 to death.

Overall survival (OS) was calculated from diagnosis of CRC to death. When the exact date of death was not available, the date of last note or referral to palliative service was taken as the date at which the patient passed.

The term line of treatment in this article was applied to any type of treatment aiming to treat or control metastatic disease. The number of lines was counted from T0. Neoadjuvant lines prior to metastatic disease were not computed in the total number of lines. When chemotherapy was given in combination with targeted therapy (e.g., FOLFOX and bevacizumab) or if maintenance treatment was given (e.g., oral capecitabine), it was considered as a single line. However, if targeted therapy (e.g., panitumumab monotherapy) or an oral systemic therapy was given alone, it was considered a separate line.

Liver resection, percutaneous ablation, and SBRT were each considered as a line of treatment irrespective of the number of lesions treated for a planned procedure. If the patient underwent a second liver resection, further ablations or SBRT (for a distinct planned volume), these were considered additional lines of treatment. DEBIRI treatment involves multiple sessions, one round of treatment was considered a single line. Surgery, ablation, radiation and embolization may be performed multiple times on separate occasions. Locoregional treatments were added to the number of chemotherapy lines which, taken together, comprised the total number of lines used to treat the patient’s metastatic disease.

Length of hospital stay (LOS) was rounded up to the number of days after DEBIRI, including the day of treatment. Readmission was considered positive if within the month following treatment. The reason for readmission was then evaluated to determine if related to the DEBIRI procedure.

Adverse events (AE) were collected based on chart review and not on patient questioning. Postembolization syndrome (PES) was not considered and AE as it was commonly encountered. AE terminology set forth by the Cancer Therapy Evaluation Program Common Terminology Criteria for Adverse Events CTCAE, version 5.0.
